# Behavioral factors associated with SARS‐CoV‐2 infection in Japan

**DOI:** 10.1111/irv.12992

**Published:** 2022-04-26

**Authors:** Takeshi Arashiro, Yuzo Arima, Hirokazu Muraoka, Akihiro Sato, Kunihiro Oba, Yuki Uehara, Hiroko Arioka, Hideki Yanai, Naoki Yanagisawa, Yoshito Nagura, Yasuyuki Kato, Hideaki Kato, Akihiro Ueda, Koji Ishii, Takao Ooki, Hideaki Oka, Yusuke Nishida, Ashley Stucky, Reiko Miyahara, Chris Smith, Martin Hibberd, Koya Ariyoshi, Motoi Suzuki

**Affiliations:** ^1^ Center for Surveillance, Immunization, and Epidemiologic Research National Institute of Infectious Diseases Tokyo Japan; ^2^ Department of Pathology National Institute of Infectious Diseases Tokyo Japan; ^3^ Faculty of Infectious and Tropical Diseases London School of Hygiene and Tropical Medicine London UK; ^4^ School of Tropical Medicine and Global Health Nagasaki University Nagasaki Japan; ^5^ CLINIC FOR Tamachi Tokyo Japan; ^6^ KARADA Internal Medicine Clinic Tokyo Japan; ^7^ Department of Pediatrics Showa General Hospital Tokyo Japan; ^8^ Department of Clinical Laboratory St. Luke's International Hospital Tokyo Japan; ^9^ Department of General Internal Medicine St. Luke's International Hospital Tokyo Japan; ^10^ Department of Clinical Laboratory, Fukujuji Hospital Japan Anti‐Tuberculosis Association Kiyose Japan; ^11^ Yanagisawa Clinic Tokyo Japan; ^12^ Shinjuku Home Clinic Tokyo Japan; ^13^ Department of Infectious Diseases, Graduate School of Medicine International University of Health and Welfare Chiba Japan; ^14^ Infection Prevention and Control Department Yokohama City University Hospital Yokohama Japan; ^15^ Department of Infectious Diseases Japanese Red Cross Medical Center Tokyo Japan; ^16^ Saitama Sekishinkai Hospital Saitama Japan; ^17^ Department of General Internal Medicine and Infectious Diseases Saitama Medical Center Saitama Japan

**Keywords:** coronavirus disease 2019 (COVID‐19), health risk behaviors, public health and social measures, risk factors, severe acute respiratory syndrome coronavirus 2 (SARS‐CoV‐2)

## Abstract

**Background:**

The relative burden of COVID‐19 has been less severe in Japan. One reason for this may be the uniquely strict restrictions imposed upon bars/restaurants. To assess if this approach was appropriately targeting high‐risk individuals, we examined behavioral factors associated with SARS‐CoV‐2 infection in the community.

**Methods:**

This multicenter case–control study involved individuals receiving SARS‐CoV‐2 testing in June–August 2021. Behavioral exposures in the past 2 weeks were collected via questionnaire. SARS‐CoV‐2 PCR‐positive individuals were cases, while PCR‐negative individuals were controls.

**Results:**

The analysis included 778 individuals (266 [34.2%] positives; median age [interquartile range] 33 [27–43] years). Attending three or more social gatherings was associated with SARS‐CoV‐2 infection (adjusted odds ratio [aOR] 2.00 [95% CI 1.31–3.05]). Attending gatherings with alcohol (aOR 2.29 [1.53–3.42]), at bars/restaurants (aOR 1.55 [1.04–2.30]), outdoors/at parks (aOR 2.87 [1.01–8.13]), at night (aOR 2.07 [1.40–3.04]), five or more people (aOR 1.81 [1.00–3.30]), 2 hours or longer (aOR 1.76 [1.14–2.71]), not wearing a mask during gatherings (aOR 4.18 [2.29–7.64]), and cloth mask use (aOR 1.77 [1.11–2.83]) were associated with infection. Going to karaoke (aOR 2.53 [1.25–5.09]) and to a gym (aOR 1.87 [1.11–3.16]) were also associated with infection. Factors not associated with infection included visiting a cafe with others, ordering takeout, using food delivery services, eating out by oneself, and work/school/travel‐related exposures including teleworking.

**Conclusions:**

We identified multiple behavioral factors associated with SARS‐CoV‐2 infection, many of which were in line with the policy/risk communication implemented in Japan. Rapid assessment of risk factors can inform decision making.

## 1 INTRODUCTION

Coronavirus disease 2019 (COVID‐19), caused by severe acute respiratory syndrome coronavirus 2 (SARS‐CoV‐2), has resulted in substantial morbidity and mortality globally.^1^ Japan has been no exception, but the relative burden of COVID‐19 after 2 years has not been as severe as in many other countries, with fewer cumulative cases and deaths relative to the population despite its aging population.^2^ Many factors may have contributed to this, such as the tireless efforts of public health centers in extensive contact tracing including backward tracing (source investigation), high mask‐wearing adherence, maintaining greater physical distance, and strict infection prevention and control measures at health‐care/long‐term care facilities.^3^, ^4^ Among these, one intriguing hypothesis is the unique policy with a focused approach targeting restaurants and bars to reduce business hours at night and prohibiting the serving of alcohol.^5^ As the Japanese government's response against COVID‐19 is based on the Act on Special Measures for Pandemic Influenza and New Infectious Diseases Preparedness and Response, Japan has declared a state of emergency several times during the course of the pandemic.^6^ However, unlike many other countries, there were no strict restrictions imposed on individual citizens such as lockdowns and obligatory curfews. Rather, there were voluntary requests to stay at home and engage in basic infection prevention measures such as proper mask wearing/hand hygiene. In comparison, restaurants and bars were ordered to suspend business if they cannot operate without serving alcohol, to stop serving alcohol, and to reduce business hours at night until 8:00 p.m. in prefectures with high transmission. Non‐compliant businesses were disclosed publicly. This specific approach towards restaurants and bars was based on individual case data and cluster investigations with a theoretical rationale that dining or drinking alcohol at restaurants and bars with others (i.e., social gatherings involving food or drinks) provides occasion to interact face to face for a prolonged period without masks and that the influence of alcohol can further lead to laxity of infection prevention measures.^7^ Also, an increase in the frequency and proportion of cases with no history of close contact^8^ made containment through cluster investigation increasingly challenging and highlighted the lack of understanding regarding risk factors for infection at the community level. These circumstances led to the need to confirm through epidemiological data, with inclusion of a control group, whether behaviors such as social gatherings are indeed risk factors for SARS‐CoV‐2 infection to inform public health policy and provide evidence‐based risk communication. Therefore, we initiated a multicenter case–control study to evaluate risk factors associated with SARS‐CoV‐2 infection, focusing on social gatherings involving food or drinks. We examined various social settings and further explored other behaviors as potential risk factors.

## 2 METHODS

### 2.1 Study design and setting

Our study, Factors Associated with SARS‐CoV‐2 Infection And The Effectiveness of COVID‐19 vaccines (FASCINATE study), is a multicenter case–control study in health‐care facilities in Japan with two objectives: (1) to elucidate risk factors associated with SARS‐CoV‐2 infection and (2) to estimate the effectiveness of COVID‐19 vaccines. Participating health‐care facilities are routinely testing outpatients using polymerase chain reaction (PCR) to diagnose SARS‐CoV‐2 infection. For this report, data from six health‐care facilities in the Kanto region (Tokyo and neighboring metropolitan prefectures) on individuals recruited during June 8–August 1, 2021, were analyzed.

### 2.2 Inclusion and exclusion criteria

Individuals who were tested for SARS‐CoV‐2 were included. Exclusion criteria were (1) individuals younger than 20 years (as alcohol drinking is illegal for these individuals), (2) individuals who did or could not consent to participate in the study, (3) individuals who could not complete the questionnaire by themselves, (4) individuals who had already participated in this study, (5) individuals who required immediate treatment, and (6) individuals with history of close contact (because an infection, if confirmed, is most likely due to this specific contact rather than exposures asked about in the questionnaire). For this report, we excluded asymptomatic individuals and individuals vaccinated at least once as COVID‐19 vaccination can influence behaviors.

### 2.3 Questionnaire and classification of cases/controls

A paper or web‐based questionnaire (according to individual preference) was administered before PCR results were available to avoid social desirability bias, where individuals who test positive may be less likely to report potentially high‐risk behaviors. The questionnaire was optimized based on a pilot study done at two sites.^9^ We defined social gathering as getting together with one or more persons that does not cohabitate with the participant. Cases were defined as PCR‐confirmed SARS‐CoV‐2 positive individuals, while controls were defined as PCR‐negative individuals.

### 2.4 Data analysis

Logistic regression to identify associations between behavioral risk factors and SARS‐CoV‐2 infection was conducted adjusting for age group, sex, presence of comorbidities, educational attainment, place of residence, past SARS‐CoV‐2 infection, health‐care facility in which SARS‐CoV‐2 testing was done, and calendar week. These potential confounders were determined *a priori*.^10^, ^11^ Data analyses were performed using STATA version 17.0.

## 3 RESULTS

### 3.1 Participant characteristics

**FIGURE 1:**
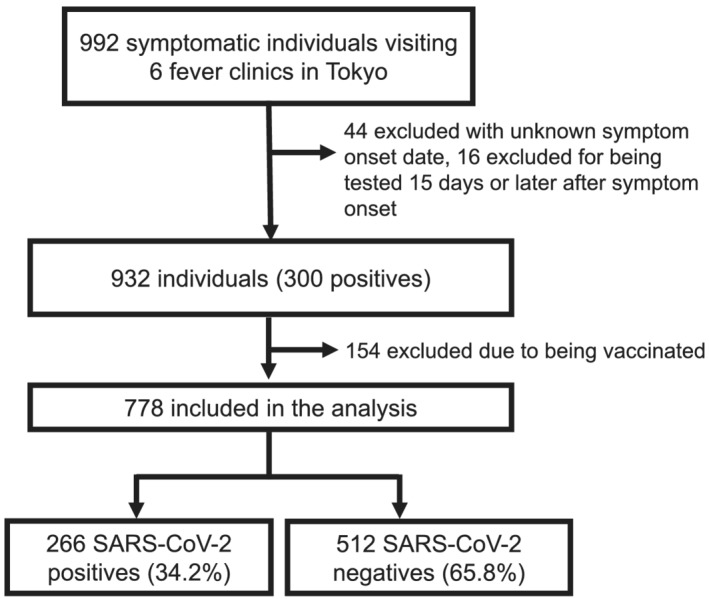
Flow diagram of study participants

A total of 992 symptomatic individuals were enrolled from six medical facilities during the study period; we excluded 44 due to unknown symptom onset, 16 due to being tested ≥15 days after symptom onset, and 154 due to being vaccinated (Figure 1). The final analysis included 778 individuals with 266 (34.2%) positive cases. The median age (interquartile range [IQR]) was 33 (27–43) years, 386 were males (49.6%), and 182 (23.4%) had comorbidities (Table 1); 758 (97.4%) were Japanese nationals and most foreigners were from East Asia.

**TABLE 1:** Demographic and clinical characteristics of the study participants

	All (n = 778)	Test‐positive (n = 266)	Test‐negative (n = 512)
Age in years, n (%)			
20–29	307 (39.5)	115 (43.2)	192 (37.5)
30–39	228 (29.3)	77 (28.9)	151 (29.5)
40–49	145 (18.6)	41 (15.4)	104 (20.3)
50–59	77 (9.9)	30 (11.3)	47 (9.2)
60–69	20 (2.6)	3 (1.1)	17 (3.3)
70–79	1 (0.1)	0 (0.0)	1 (0.2)
Sex, n (%)			
Male	386 (49.6)	141 (53.0)	245 (47.9)
Female	392 (50.4)	125 (47.0)	267 (52.2)
Educational attainment, n (%); missing = 6			
Middle school or less	10 (1.3)	5 (1.9)	5 (1.0)
High school	130 (16.8)	56 (21.1)	74 (14.6)
Junior college/technical college	171 (22.2)	58 (21.9)	113 (22.3)
Undergraduate or graduate school	461 (59.7)	146 (55.1)	315 (62.1)
Place of residence, n (%); missing = 2			
Home	760 (97.9)	257 (97.4)	503 (98.2)
Hospital or long‐term care facility	2 (0.3)	1 (0.4)	1 (0.2)
Dormitory or other	14 (1.8)	6 (2.3)	8 (1.6)
Comorbidities, n (%)			
Yes	182 (23.4)	71 (26.7)	111 (21.7)
No	596 (76.6)	195 (73.3)	401 (78.3)
Smoking, n (%); missing = 2			
Never smoker	441 (56.8)	134 (50.8)	307 (60.0)
Past smoker	176 (22.7)	57 (21.6)	119 (23.2)
Current smoker	159 (20.5)	73 (27.7)	86 (16.8)
Days from onset to SARS‐CoV‐2 test^a^; missing = 4			
	2 (1–3)	2 (1–3)	2 (1–3)
SARS‐CoV‐2 diagnostic test in the past month, n (%); missing = 10			
Yes	118 (15.4)	49 (18.7)	69 (13.6)
No	650 (84.6)	213 (81.3)	437 (86.4)
Past SARS‐CoV‐2 infection, n (%); missing = 18			
Yes	13 (1.7)	3 (1.2)	10 (2.0)
No	747 (98.3)	255 (98.8)	492 (98.0)

^a^ Median (interquartile range).

### 3.2 Factors related to 3Cs and five situations in the past 2 weeks

Since early in the pandemic, the Japanese government has been promoting avoidance of the “3Cs,” representing (1) closed spaces, (2) crowded places, and (3) close‐contact settings, which are considered high‐risk based on characteristics of early clusters.^12^ These “3Cs” were easy for the public to remember and the World Health Organization also began promoting this message.^13^ Additionally, since fall 2020, the government started to promote avoidance of “five situations,” namely, (1) social gatherings that include alcohol consumption, (2) large group gatherings that involve eating and/or drinking for an extended period of time, (3) conversing without a mask, (4) cohabitation in small living quarters, and (5) relocating to a different area.^12^ We first examined factors among the above that could be measured (Table 2). Those who attended large gatherings that involve eating and/or drinking for an extended period of time in the past 2 weeks had particularly higher odds of infection compared with those who did not (adjusted odds ratio [aOR] 2.36 [1.38–4.05]).

**TABLE 2:** Association of SARS‐CoV‐2 infection with various activities/situations

	Test‐positive, n (%)	Test‐negative, n (%)	Crude odds ratio (95% CI)	Adjusted odds ratio (95% CI)^a^
Having a conversation at a close distance (within arm's reach)				
No	142 (53.4)	283 (55.3)	1	1
Yes	124 (46.6)	229 (44.7)	1.08 (0.80–1.45)	0.92 (0.65–1.30)
Closed spaces with poor ventilation/air exchange				
No	225 (84.6)	460 (89.8)	1	1
Yes	41 (15.4)	52 (10.2)	1.61 (1.04–2.50)	1.24 (0.76–2.03)
Large gatherings that involve eating and/or drinking for an extended period of time				
No	221 (83.1)	471 (92.0)	1	1
Yes	45 (16.9)	41 (8.0)	2.34 (1.49–3.68)	2.36 (1.38–4.05)
Crowded places				
No	189 (71.1)	378 (73.8)	1	1
Yes	77 (29.0)	134 (26.2)	1.15 (0.83–1.60)	1.03 (0.71–1.50)
Cohabitation in small living quarters				
No	250 (94.0)	472 (92.2)	1	1
Yes	16 (6.0)	40 (7.8)	0.76 (0.41–1.38)	0.77 (0.39–1.53)
Frequency of social gatherings attended that involved eating/drinking				
0 (did not attend)	75 (28.9)	208 (41.8)	1	1
1	38 (14.6)	85 (17.1)	1.24 (0.78–1.97)	1.37 (0.81–2.31)
2	42 (16.2)	73 (14.7)	1.60 (1.00–2.53)	1.60 (0.94–2.72)
≥3	105 (40.4)	132 (26.5)	2.21 (1.53–3.19)	2.00 (1.31–3.05)
Presence or absence of alcohol in social gatherings that involved eating/drinking				
Did not attend	74 (28.8)	207 (41.8)	1	1
No alcohol	38 (14.8)	111 (22.4)	0.96 (0.61–1.51)	0.93 (0.56–1.55)
With alcohol	145 (56.4)	177 (35.8)	2.29 (1.62–3.23)	2.29 (1.53–3.42)
Location of social gatherings attended that involved eating/drinking				
Did not go out to eat	75 (31.9)	208 (43.5)	1	1
Only at home	16 (6.8)	21 (4.4)	2.11 (1.05–4.26)	2.10 (0.92–4.77)
Restaurants/bars^b^	134 (57.0)	239 (50.0)	1.55 (1.11–2.18)	1.55 (1.04–2.30)
Outdoors/parks^c^	10 (4.3)	10 (2.1)	2.77 (1.11–6.93)	2.87 (1.01–8.13)
Time of day of social gatherings attended that involved eating/drinking				
Did not go out to eat	75 (28.9)	208 (41.8)	1	1
Daytime only	20 (7.7)	76 (15.3)	0.73 (0.42–1.28)	0.76 (0.41–1.42)
Evening/night	165 (63.5)	214 (43.0)	2.14 (1.53–2.98)	2.07 (1.40–3.04)
Visiting a cafe with others				
No	140 (58.1)	306 (64.3)	1	1
Yes	101 (41.9)	170 (35.7)	1.30 (0.95–1.78)	1.08 (0.75–1.57)
Ordering takeout				
No	160 (64.5)	291 (60.8)	1	1
Yes	88 (35.5)	188 (39.3)	0.85 (0.62–1.17)	0.80 (0.56–1.15)
Food delivery				
No	149 (58.7)	309 (63.5)	1	1
Yes	105 (41.3)	178 (36.6)	1.22 (0.90–1.67)	1.40 (0.97–2.01)
Eating out by oneself				
No	150 (59.1)	281 (57.6)	1	1
Yes	104 (40.9)	207 (42.4)	0.94 (0.69–1.28)	0.79 (0.55–1.13)
Maximum number of people attended including oneself				
Did not go out to eat/drink or to a cafe	61 (28.6)	169 (39.2)	1	1
<5 people	112 (52.6)	213 (49.4)	1.46 (1.00–2.11)	1.31 (0.86–2.00)
≥5 people	40 (18.8)	49 (11.4)	2.26 (1.36–3.77)	1.81 (1.00–3.30)
Maximum time spent				
Did not go out to eat/drink or to a cafe	61 (25.4)	169 (36.3)	1	1
<2 h	51 (21.3)	118 (25.3)	1.20 (0.77–1.86)	1.01 (0.61–1.65)
≥2 h	128 (53.3)	179 (38.4)	1.98 (1.37–2.87)	1.76 (1.14–2.71)
Mask wearing (study participant)				
Did not go out to eat/drink or to a cafe	61 (25.0)	169 (36.4)	1	1
Wore at all times except when eating/drinking	28 (11.5)	72 (15.5)	1.08 (0.64–1.82)	0.96 (0.53–1.73)
Took mask off when food/drink was served	100 (41.0)	185 (39.9)	1.50 (1.02–2.19)	1.29 (0.84–2.00)
Did not wear one/took mask off when seated	55 (22.5)	38 (8.2)	4.01 (2.42–6.66)	4.18 (2.29–7.64)
Mask wearing (others)				
Did not go out to eat/drink or to a cafe	61 (26.2)	169 (37.1)	1	1
Wore at all times except when eating/drinking	20 (8.6)	51 (11.2)	1.09 (0.60–1.97)	0.96 (0.49–1.86)
Took mask off when food/drink was served	89 (38.2)	190 (41.8)	1.30 (0.88–1.91)	1.12 (0.72–1.74)
Did not wear one/took mask off when seated	63 (27.0)	45 (9.9)	3.88 (2.40–6.28)	3.74 (2.13–6.55)

^a^ Adjusted for age group, sex, presence of comorbidities, educational attainment, place of residence, past SARS‐CoV‐2 infection, health‐care facility, and calendar week.

^b^ Individuals may or may not have history of gathering at home.

^c^ Individuals may or may not have history of gathering at home, restaurants, and bars.

### 3.3 Association between social gatherings with food/drinks in various settings in the past 2 weeks and SARS‐CoV‐2 infection

We further examined the association between social gatherings that involve eating and/or drinking in various settings and SARS‐CoV‐2 infection (Table 2). The odds of infection increased with increased frequency of social gatherings; those who attending social gatherings three or more times had higher odds of infection compared with those who did not (aOR 2.00 [95% CI 1.31–3.05]). We examined this association in detail, specifically by presence of alcohol, location of gathering, and time of day. The odds of infection were substantially higher among individuals who attended social gatherings with alcohol at least once compared with those who did not (aOR 2.29 [95% CI 1.53–3.42]). Social gatherings without alcohol were not associated with infection. When we compared the location of social gatherings, the odds of infection were higher among individuals who had gatherings only at home (aOR 2.10 [95% CI 0.92–4.77]), visited bars or restaurants (aOR 1.55 [95% CI 1.04–2.30]), and had gatherings involving food/drinks outdoors/at parks (aOR 2.87 [95% CI 1.01–8.13]), all compared with those who did not. Moreover, the odds of infection among individuals who attended social gatherings at night were double that of those who did not (aOR 2.07 [95% CI 1.40–3.04]). Attending social gatherings only in the daytime was not associated with infection.

### 3.4 Association between other behaviors related to food/drinks in the past 2 weeks and SARS‐CoV‐2 infection

To compare the above findings on social gatherings that involve eating and/or drinking, we examined whether other behaviors related to food/drinks were associated with SARS‐CoV‐2 infection (Table 2). Unlike social gatherings, the odds of infection were not higher among individuals who visited a cafe with others (aOR 1.08 [95% CI 0.75–1.57]), ordered takeout (aOR 0.80 [95% CI 0.56–1.15]), used food delivery services (aOR 1.40 [95% CI 0.97–2.01]), and ate out by oneself (aOR 0.79 [95% CI 0.54–1.13]), all compared with those who did not.

### 3.5 Association between size, duration, and mask wearing among participants of social gatherings in the past 2 weeks and SARS‐CoV‐2 infection

The government requested individuals attending social gatherings involving food/drinks to limit these gatherings to five people and to less than 2 hours and to consider use of masks except for when consuming food/drinks.^14^ We assessed whether these factors were indeed associated with infection (Table 2). Specifically, those who attended a social gathering involving food/drinks and/or went to a cafe with others in the past 2 weeks were asked how many people attended the gathering, how long the gathering continued at maximum, and when the attendees had their masks on (cafe use was included here as we hypothesized that it also provides occasion to talk face to face for a prolonged period without masks). The odds of infection were higher among individuals who attended gatherings of five or more people (aOR 1.81 [95% CI 1.00–3.30]) and for individuals who attended for 2 hours or longer (aOR 1.76 [95% CI 1.14–2.71]), both compared with those who did not attend gatherings. Regarding mask wearing, the odds of infection were higher among those who did not wear a mask or took it off when seated (aOR 4.18 [95% CI 2.29–7.64]). This association was similar when the mask‐wearing practices of other attendees at the gathering was assessed (aOR 3.74 [95% CI 2.13–6.55]).

### 3.6 Association between type of mask used in the past 2 weeks and SARS‐CoV‐2 infection

Three types of masks are mainly used among the public in Japan: medical/surgical masks, cloth masks, and polyurethane masks. The Japanese government recommends use of medical/surgical masks rather than cloth or polyurethane masks based on a computer simulation model,^15^ but epidemiological data were lacking. Therefore, we examined the association between mask type and infection (Table 3). The odds of infection were higher among individuals who used cloth masks (aOR 1.77 [95% CI 1.00–3.30]) and slightly higher among individuals who used polyurethane masks (aOR 1.47 [95% CI 0.91–2.38]), both compared with those who used medical/surgical masks. When we stratified dichotomously by whether the participants attended social gatherings or visited a cafe with others, the aforementioned association between infection and cloth or polyurethane mask use compared with medical/surgical mask use was only present among individuals who attended social gatherings or visited a cafe with others.

**TABLE 3:** Association of SARS‐CoV‐2 infection with type of mask used

	Test‐positive, n (%)	Test‐negative, n (%)	Crude odds ratio (95% CI)	Adjusted odds ratio (95% CI)^a^
Type of mask used regularly				
Medical/surgical mask	134 (55.6)	340 (69.4)	1	1
Cloth mask	53 (22.0)	82 (16.7)	1.64 (1.10–2.44)	1.77 (1.11–2.83)
Polyurethane mask	53 (22.0)	68 (13.9)	1.97 (1.31–2.98)	1.47 (0.91–2.38)
No mask	1 (0.4)	0 (0.0)	N/A	N/A
Type of mask used regularly (individuals who *did not* report attending social gathering or visiting a cafe with others)				
Medical/surgical mask	33 (61.1)	114 (68.7)	1	1
Cloth mask	11 (20.4)	32 (19.3)	1.19 (0.54–2.61)	1.48 (0.51–4.29)
Polyurethane mask	10 (18.5)	20 (12.1)	1.73 (0.74–4.05)	0.75 (0.24–2.37)
No mask	0 (0.0)	0 (0.0)	N/A	N/A
Type of mask used regularly (individuals who reported attending social gathering or visiting a cafe with others)				
Medical/surgical mask	98 (53.9)	219 (70.9)	1	1
Cloth mask	42 (23.1)	46 (14.9)	2.04 (1.26–3.30)	2.03 (1.16–3.56)
Polyurethane mask	41 (22.5)	44 (14.2)	2.08 (1.28–3.39)	1.62 (0.91–2.89)
No mask	1 (0.6)	0 (0.0)	N/A	N/A

^a^ Adjusted for age group, sex, presence of comorbidities, educational attainment, place of residence, past SARS‐CoV‐2 infection, health‐care facility, and calendar week.

### 3.7 Association between behaviors not related to food/drinks in the past 2 weeks and SARS‐CoV‐2 infection

We next looked at behaviors that do not involve eating/drinking that are considered to be risk factors based on case/cluster investigations in Japan^7^ (Table 4). The odds of infection were not higher among individuals who attended indoor events and gatherings (aOR 1.45 [95% CI 0.76–2.76]) and outdoor events and gatherings (aOR 1.34 [95% CI 0.67–2.69]), both compared with those who did not. The odds of infection were lower among individuals who went to department stores and shopping malls (aOR 0.64 [95% CI 0.45–0.91]) compared with those who did not. In contrast, the odds of infection were higher among individuals who attended karaoke with others (aOR 2.53 [95% CI 1.25–5.09]) and individuals who visited a gym (aOR 1.87 [95% CI 1.11–3.16]), both compared with those who did not.

**TABLE 4:** Association of SARS‐CoV‐2 infection with behaviors other than going out to eat/drink

	Test‐positive, n (%)	Test‐negative, n (%)	Crude odds ratio (95% CI)	Adjusted odds ratio (95% CI)^a^
Indoor events/gathering^b^				
No	219 (90.9)	432 (92.1)	1	1
Yes	22 (9.1)	37 (7.9)	1.17 (0.68–2.04)	1.45 (0.76–2.76)
Outdoor events/gathering^b^				
No	221 (91.7)	435 (94.4)	1	1
Yes	20 (8.3)	26 (5.6)	1.51 (0.83–2.77)	1.34 (0.67–2.69)
Department stores and shopping malls				
No	116 (46.8)	189 (39.1)	1	1
Yes	132 (53.2)	294 (60.9)	0.73 (0.54–1.00)	0.64 (0.45–0.91)
Karaoke with others				
No	227 (90.4)	463 (96.1)	1	1
Yes	24 (9.6)	19 (3.9)	2.58 (1.38–4.80)	2.53 (1.25–5.09)
Gym				
No	212 (83.5)	432 (89.8)	1	1
Yes	42 (16.5)	49 (10.2)	1.75 (1.12–2.72)	1.87 (1.11–3.16)
Work/school				
No	32 (12.2)	77 (15.2)	1	1
Yes	231 (87.8)	430 (84.8)	1.29 (0.83–2.01)	1.01 (0.60–1.69)
Work/school full time^c^				
Part time	28 (12.5)	65 (15.4)	1	1
Full time	196 (87.5)	358 (84.6)	1.27 (0.79–2.04)	1.11 (0.65–1.90)
Use trains to commute^c^				
No	79 (34.2)	133 (30.9)	1	1
Yes	152 (65.8)	297 (69.1)	0.86 (0.61–1.21)	0.84 (0.57–1.24)
Frequency of teleworking/attending online classes^c^ ^,^ ^d^				
0%	98 (51.3)	180 (51.3)	1	1
25%	34 (17.8)	45 (12.8)	1.39 (0.83–2.31)	1.17 (0.63–2.17)
50%	17 (8.9)	46 (13.1)	0.68 (0.37–1.25)	0.71 (0.36–1.43)
75%	21 (11.0)	30 (8.6)	1.29 (0.70–2.37)	1.55 (0.78–3.10)
Almost 100%	21 (11.0)	50 (14.3)	0.77 (0.44–1.36)	0.99 (0.51–1.91)
Residing/visiting an urban location^e^				
Never	15 (5.9)	55 (11.0)	1	1
Residing an urban location	180 (70.6)	326 (65.5)	2.02 (1.11–3.69)	3.46 (1.52–7.84)
Visiting an urban location	60 (23.5)	117 (23.5)	1.88 (0.98–3.60)	2.43 (1.11–5.33)
Travel				
No travel	224 (92.2)	418 (94.1)	1	1
Business travel	5 (2.1)	12 (2.7)	0.78 (0.27–2.23)	1.05 (0.32–3.47)
Non‐business travel	14 (5.8)	14 (3.2)	1.87 (0.87–3.98)	1.56 (0.65–3.73)

^a^ Adjusted for age group, sex, presence of comorbidities, educational attainment, place of residence, past SARS‐CoV‐2 infection, health‐care facility, and calendar week.

^b^ Gatherings include events, social groups, and school extracurricular activities.

^c^ Restricted to individuals with work and/or school.

^d^ Restricted to individuals who work full time.

^e^ Surrounding areas of city centers/major train stations.

### 3.8 Association between behaviors related to work/school and travel in the past 2 weeks and SARS‐CoV‐2 infection

We lastly examined whether behaviors related to work/school were associated with SARS‐CoV‐2 infection (Table 4). The odds of infection were not higher among individuals who have work/school (aOR 1.01 [95% CI 0.60–1.69]) and who attended work/school full time (aOR 1.11 [95% CI 0.65–1.90]), compared with those who did not or who attended work/school part time. As millions of people commute each day in metropolitan prefectures in crowded trains, many were afraid of being infected on these trains, but the odds of infection were not higher among those who used trains to commute (aOR 0.84 [95% CI 0.57–1.24]). Teleworking was also encouraged by the government, but the odds of infection were not lower among individuals who teleworked/attended online classes almost 100% of the time (aOR 0.99 [95% CI 0.51–1.91]), compared with those who did not. When asked about travel‐related factors, the odds of infection were higher among individuals who reside in an urban location (aOR 3.46 [95% CI 1.52–7.84]) or visited an urban location (aOR 2.43 [95% CI 1.11–5.33]), compared with those who did not, respectively. In contrast, the odds of infection were not higher among individuals who traveled on business (aOR 1.05 [95% CI 0.32–3.47]) or for non‐business purposes (aOR 1.56 [95% CI 0.65–3.73]), compared with those who did not travel.

## 4 DISCUSSION

In this multicenter case–control study, we investigated the association between various behavioral factors and SARS‐CoV‐2 infection in the community setting. First, we found that attending social gatherings with food/drinks was associated with SARS‐CoV‐2 infection. We strengthened our findings and those of previous reports^16^, ^17^, ^18^ by showing the association in a dose‐dependent manner, with the odds of infection increasing with increasing frequency of social gatherings in the past 2 weeks. We also investigated the details of specific settings of social gatherings that were associated with infection. First, attending social gatherings with alcohol was associated with infection. When we examined the location of gatherings, attending gatherings at restaurants or bars was associated with infection. This finding was consistent with previous ecological/modeling and case–control studies where going to restaurants or bars was associated with infection.^16^, ^17^, ^18^ As there were strict restrictions imposed upon restaurants and bars in Japan, there was a concern that people may choose to have gatherings at home or out on public streets/parks, with 10% of young people reporting that they had done the latter.^19^ Indeed, individuals who had social gatherings exclusively at home had higher odds of infection, and attending gatherings outdoors or at parks was associated with infection. Attending a gathering at night was also associated with infection. The reason may be that social gatherings at night tend to be longer in duration, and individuals may become more intoxicated and care less about infection prevention measures. In contrast to the findings on social gatherings, ordering takeout, using food delivery services, and eating out by oneself were not associated with infection. This is expected as these behaviors would not substantially increase contact with others; our findings provide opportunity for the food industry to sustain its business. Details about how these gatherings took place also mattered; attending a gathering with five or more people or gathering lasting 2 hours or longer was associated with infection. Not wearing a mask or taking it off when seated at the gathering was also associated with infection, supporting the idea of “mask‐dining” (taking off the mask only when putting food in the mouth or sipping drinks and keeping the mask on while talking, while waiting for food/drinks to be served, and after finishing meals), which has been recommended by the government when at restaurants and bars. On a related note, regular use of cloth or polyurethane masks was associated with infection, specifically among individuals who attended social gatherings or visited a cafe with others. In addition to source control, the association here suggested the protective effect of medical/surgical masks,^20^ and individuals who engage in high‐risk behaviors may benefit from wearing medical/surgical masks. We could not evaluate associations with any mask use, as the mask‐wearing adherence was high.

We identified some factors unrelated to social gatherings, namely, karaoke and gym use, which are also known to be hotspots for clusters.^7^ Shopping at department stores and shopping malls was negatively associated with infection. This may have been because individuals in metropolitan areas visit department stores to buy products for use at home, although there have been reported clusters in these settings.^21^ Unlike in previous reports from the United States and France,^18^, ^22^ we did not find a negative association between teleworking and infection, which may reflect the difference in the magnitude of epidemics or the amount of physical contact at the work place.

These findings collectively indicate that, although epidemiological evidence was scarce at the time of implementation of the targeted policies in Japan, the policies appropriately targeted high‐risk individuals and activities/situations. Our results highlight the importance of rapidly assessing and identifying modifiable behavioral risk factors for SARS‐CoV‐2 infection and of informing risk communication and shaping public health policy with targeted approaches tailored to each country's situation, in addition to universal recommendations. Targeted approaches can have substantial financial consequences to individuals, so appropriate social security and welfare policies should be paired with restrictions imposed.

This study had several limitations. First, biases inherent in observational studies such as recall bias and residual confounding are possible. Second, controls may have been infected with other viruses due to similar exposures, which can underestimate the odds ratio (Supporting Information). Third, our primary analyses were complete case analyses. However, when we did sensitivity analyses to impute missing exposure variables in each analysis, findings were similar. Finally, identified risk factors may be country/region/culture‐specific and time‐dependent due to changes in COVID‐19‐related policies and behaviors, as well as emergence of SARS‐CoV‐2 variants.

In conclusion, we identified multiple behavioral factors associated with SARS‐CoV‐2 infection, particularly in various settings of social gatherings. These factors may be country/culture‐specific and time‐dependent due to changes in COVID‐19‐related policies and behaviors, so continuous monitoring in various settings is important to inform decision making.

## AUTHOR CONTRIBUTIONS

**Takeshi Arashiro:**Conceptualization; data curation; formal analysis; investigation; methodology; project administration; resources; software; visualization; funding acquisition. **Yuzo Arima:** Conceptualization; formal analysis; methodology; resources; supervision. **Hirokazu Muraoka:** Investigation. **Akihiro Sato:** Investigation. **Kunihiro Oba:** Investigation. **Yuki Uehara:** Investigation. **Hiroko Arioka:** Investigation. **Hideki Yanai:** Investigation. **Naoki Yanagisawa:** Investigation. **Yoshito Nagura:** Investigation. **Yasuyuki Kato:** Investigation. **Hideaki Kato:** Investigation. **Akihiro Ueda:** Investigation. **Koji Ishii:** Investigation. **Takao Ooki:** Investigation. **Hideaki Oka:** Investigation. **Yusuke Nishida:** Investigation. **Ashley Stucky:** Formal analysis; investigation; visualization. **Reiko Miyahara:** Formal analysis; methodology. **Chris Smith:** Conceptualization; formal analysis; methodology; supervision. **Martin Hibberd:** Conceptualization; formal analysis; methodology; supervision. **Koya Ariyoshi:** Conceptualization; formal analysis; methodology; supervision. **Motoi Suzuki:** Conceptualization; formal analysis; funding acquisition; methodology; supervision.

## Supporting information


**Data S1.**Supporting Information

## Data Availability

Individual‐level data of patients included in this manuscript after de‐identification are considered sensitive and will not be shared. The study methods and statistical analyses are all described in detail in Section 2 and throughout the manuscript.
