# Supplementary material for: Behavioral factors associated with SARS‐CoV‐2 infection in Japan
Source: Influenza Other Respir Viruses. 2022 Apr 26;16(5):952–61. doi: 10.1111/irv.12992 (PMC9111610; doi:10.1111/irv.12992)
Supplement: Supplementary file 1 — Data S1.Supporting Information [file IRV-16-952-s001.docx]

**Supporting Information**

*COVID-19 situation in Japan during the study period*

During this period, transmission intensity (i.e. notification rate) was relatively high and cases reported increased from 20 to 160 per 100 000 population in Tokyo, with a state of emergency in effect during June 8-20 as well as July 12-August 1, and a semi-state of emergency during June 21-July 11. The study period was the replacement period from the alpha variant to the delta variant in the Kanto region.

*Symptomatic infection*

We considered individuals as symptomatic if they had any of the following: fever above 37.5°C, malaise, chills, joint pain, headache, runny nose, cough, sore throat, shortness of breath, gastrointestinal symptoms (e.g., vomiting, diarrhea, stomach ache), and loss of taste/smell.

*Definition of comorbidities*

Comorbidities included any one of the following: hypertension, heart disease, diabetes mellitus, obesity, kidney disease, asthma, chronic obstructive pulmonary disease, cancer, immunodeficiency, and immunosuppressant use.

*Choice of controls*

A test-negative design (individuals tested for SARS-CoV-2 with negative results serving as controls) has been established and utilized extensively for the evaluation of vaccine effectiveness for infectious diseases such as influenza and COVID-19; this is because of the antigen specificity of the vaccines.^1-5^ As for risk factor analysis, it is possible that test-negative individuals also share some risk factors with the cases as the controls are also symptomatic. Although ideal controls would be individuals who are randomly selected who test negative, and arise from the same population as the cases, this was not possible for us due to feasibility. We considered utilizing other additional controls while formulating the protocol. One potential method was to have an accompanying person (AP) as a control, but individuals getting tested for SARS-CoV-2 who are not severely ill often go to health-care facilities alone to keep the AP from becoming a close contact, so it was not feasible in our setting (APs are usually not allowed in the medical facilities for this reason). Another option was individuals who are getting tested with no symptoms and are not a close contact of a case. However, there is an issue of why these individuals are getting tested in the first place. In Japan, people who get tested despite being asymptomatic are usually at high risk of exposure by nature of their occupation (e.g. health-care workers and other specific occupations) or behaviors (individuals who engage in high-risk recreational behaviors regularly, individuals who are constantly worried about getting infected [e.g. mysophobia], individuals travelling overseas, etc.). Asymptomatic individuals getting tested for such reasons would not serve as a representative sample of the source population that gave rise to the cases. The final option was to have traditional hospital controls who visit other departments such as surgery and orthopedics. However, these would not be appropriate since these individuals are quite older on average and have a very skewed distribution for the factors that we are and are not interested in, including potential confounding factors. Also, there are no such departments in many of our study sites as they are small clinics. Often, it can be very challenging to match the traits of cases with controls in a case-control study.^6^ We concluded that the baseline and demographic traits among cases and controls would be most similar with a test-negative approach, as the two groups would be sourced from those presenting to the same medical facilities for testing (e.g. health-seeking behaviors)—and as long as participants complete the questionnaire before receiving their test results, the influence of social desirability bias should be minimal. Also, if controls were infected with other viruses due to similar exposures, the odds ratio for SARS-CoV-2 infection would be an underestimate of the true association. In other words, our design would detect differences in magnitude of a particular risk factor or risk factors that would be specific for COVID-19. In fact, even though many respiratory pathogens (influenza virus, *Streptococcus pneumoniae*, etc.) were present at extremely low levels during the study period, at least partially due to social and public health measures, we have continued to see repeated SARS-CoV-2 epidemics. This suggests that SARS-CoV-2 has unique features that allow it to circulate even under strict public health and social measures. Finally, the threshold for testing was low for SARS-CoV-2 in Japan at the time of the study and having any one of the very broad spectrum of signs/symptoms would trigger testing and hence inclusion in the study, not just individuals with respiratory symptoms. Therefore, at the start of the investigation, we expected to identify at least some risk factors identified in other studies that utilized similar methods to elucidate risk factors.^7-9^

*Sample size calculation*

Assuming 10% positivity (based on data when the study was planned),^8^ 30-50% of controls having the exposures of interest, a two-tailed significance level of 5%, and 80% power, enrollment of approximately 70-80 cases and 700-800 controls was needed to detect a minimal odds ratio of 2. We planned to continue enrollment even after reaching this target to allow for sub-analysis and continued assessment of risk factors that may be time-varying.

**References**

1. Dagan N, Barda N, Kepten E, et al. BNT162b2 mRNA Covid-19 vaccine in a nationwide mass vaccination setting. *N Engl J Med*. 2021;384:1412–1413.
2. Dean NE, Hogan JW, Schnitzer ME. Covid-19 vaccine effectiveness and the test-negative design. *N Engl J Med*. 2021;385:1431–1433.
3. Jackson ML, Nelson JC. The test-negative design for estimating influenza vaccine effectiveness. *Vaccine*. 2013;31:2165–2168.
4. Lopez Bernal J, Andrews N, Gower C, et al. Effectiveness of Covid-19 vaccines against the B.1.617.2 (Delta) variant. *N Engl J Med*. 2021;385:585–594.
5. Sullivan SG, Feng S, Cowling BJ. Potential of the test-negative design for measuring influenza vaccine effectiveness: a systematic review. Expert Rev *Vaccines*. 2014;13:1571–1591.
6. Vandenbroucke JP, Brickley EB, Vandenbroucke-Grauls CMJE, Pearce N. A test-negative design with additional population controls can be used to rapidly study causes of the SARS-CoV-2 epidemic. *Epidemiology*. 2020;31:836–843.
7. Fisher KA, Tenforde MW, Feldstein LR, et al. Community and close contact exposures associated with COVID-19 among symptomatic adults ≥18 years in 11 outpatient health care facilities - United States, July 2020. *MMWR Morb Mortal Wkly Rep*. 2020;69:1258–1264.
8. Fisher KA, Olson SM, Tenforde MW, et al. Telework before illness onset among symptomatic adults aged ≥18 years with and without COVID-19 in 11 outpatient health care facilities - United States, July 2020. *MMWR Morb Mortal Wkly Rep*. 2020;69:1648–1653.
9. Tenforde MW, Fisher KA, Patel MM. Identifying COVID-19 risk through observational studies to inform control measures. *JAMA*. 2021;325:1464–1465.
